# Hydrological Response to Precipitation and Human Activities—A Case Study in the Zuli River Basin, China

**DOI:** 10.3390/ijerph15122780

**Published:** 2018-12-07

**Authors:** Chenlu Huang, Qinke Yang, Weidong Huang, Junlong Zhang, Yuru Li, Yucen Yang

**Affiliations:** 1 College of Urban and Environment Sciences, Northwest University, Xi’an 710127, China; nwuhcl@163.com (C.H.); liyuru@stumail.nwu.edu.cn (Y.L.); 2 Hydrology and Water Resources Bureau of Gansu Province, Lanzhou 730000, China; gsdxhwd@163.com; 3 College of Geography and Environment, Shandong Normal University, Jinan 250358, China; junlongzhangcq@hotmail.com; 4 First Geographic Information Mapping Institute, National Administration of Surveying, Mapping and Geoinformation, Xi’an 710127, China; yangyucen104@163.com

**Keywords:** Zuli River Basin, precipitation, runoff, sediment discharge, soil conservation measure

## Abstract

Precipitation and human activities are two essential forcing dynamics that influence hydrological processes. Previous research has paid more attention to either climate and streamflow or vegetation cover and streamflow, but rarely do studies focus on the impact of climate and human activities on streamflow and sediment. To investigate those impacts, the Zuli River Basin (ZRB), a typical tributary basin of the Yellow River in China, was chosen to identify the impact of precipitation and human activities on runoff and sediment discharge. A double mass curve (DMC) analysis and test methods, including accumulated variance analysis, sequential cluster, Lee-Heghnian, and moving *t*-test methods, were utilized to determine the abrupt change points based on data from 1956 to 2015. Correlation formulas and multiple regression methods were used to calculate the runoff and sediment discharge reduction effects of soil conservation measures and to estimate the contribution rate of precipitation and soil conservation measures to runoff and sediment discharge. Our results show that the runoff reduction effect of soil conservation measures (45%) is greater than the sediment discharge reduction effect (32%). Soil conservation measures were the main factor controlling the 74.5% and 75.0% decrease in runoff and sediment discharge, respectively. Additionally, the contribution rate of vegetation measures was higher than that of engineering measures. This study provides scientific strategies for water resource management and soil conservation planning at catchment scale to face future hydrological variability.

## 1. Introduction

Climate variability and human activity have been recognized as being important in the changes of river hydrological processes [1,2], and baseflow and sediment load are all important issues in water resource management [3,4,5], particularly in the arid and semi-arid regions of western China. For example, Li, et al. [6] chose the Wuding River Basin as a typical catchment for assessing the impact of climate variability and human activities on streamflow. They found that the reduction of streamflow due to changes in soil conservation measures was much larger than those due to precipitation variations. Huang and Zhang [7] calculated the hydrological responses to conservation practices in the Jialuhe River catchment without considering the impact of changes in precipitation. He reported that the annual surface runoff decreased by 32% due to tree plantations and that the runoff decreased rapidly in summer and decreased slightly in winter. In general, runoff and sediment loads have been influenced by global climate change and human activities and have changed dramatically within large river basins [8]. In the context of climate shift and human intervention, assessing the impacts of precipitation and soil conservation on the runoff and sediment discharge in the Yellow River Basin is important for better water resource management and planning soil conservation measures. 

The combination of precipitation and human activities leads to variations of runoff and sediment load in the Yellow River Basin [9,10]. For example, on the Loess Plateau, where water resources are scarce, precipitation is the main climatic factor that directly controls the yield of runoff and sediment load [11,12]. The amount of incoming water and sediment in the basin mainly depends on the amount of previous rainfall, precipitation intensity and total precipitation [13,14]. Over the past studies, lots of researchers have been concerned with evaluating the streamflow changes of climate and land cover [15], while paying little attention to sediment load changes and other human activities, except land cover. Human activities, especially soil conservation measures (i.e., terracing, afforestation, and construction of sediment trapping dams), are significantly affecting the variations of runoff and sediment load [16]; therefore, controlling the soil erosion [17,18] and soil and water loss in the basin plays an important role in reducing runoff and sediment [7]. On the Loess Plateau, large-scale development of farmland had been conducted in most areas since the 1950s, resulting in a substantial increase in the area occupied by level terraces [19]. After the 1980s, small watershed management projects and measures such as the establishment of tree plantations were vigorously implemented. These measures have changed the underlying surface conditions and have had a profound impact on the erosion and sediment load [20]. Since 2000, the vegetation condition has been improved considerably. During the same period, the runoff and sediment in the Yellow River Basin had also decreased substantially [21,22]. Additionally, streamflow, which provides data that serve as an important source for hydrological analysis and the evaluation of water resources, has also exhibited tremendous variations as a result of the construction of reservoirs and the implementation of irrigation project [14,23]. 

Extensive research has been conducted on how to model and forecast the rainfall-runoff [24,25], and quantitatively analyze the impacts of climate change and human activities on the runoff and sediment load of a river. The study by Gao, et al. [26] indicates that human activities have been the main factor that led to the decrease of runoff and sediment load in the Wuding River Basin in the past 60 years. Wang, et al. [27] proposed a method (the Slope Changing Ratio of Cumulative Quantity) suitable for arid and semi-arid regions and found that the relative contribution rate of human activities to the reduction of runoff in the Huangfuchuan drainage basin is much larger than the contribution rate associated with precipitation. Zhang, et al. [28] found that implementation of soil conservation measures is the main reason for the decrease of runoff and sediment yield through his analysis of the runoff and sediment reduction effect in the Wuding River Basin. Among those measures, the impact of vegetation on runoff and engineering on sediment yield are important. However, most of the previous studies have focused on the average annual precipitation but seldom considered the effect of flood season precipitation. In addition, during his research on the effect of water and sediment reduction in the Zuli River Basin (ZRB), Zhao [29] used the measured data from hydrologic and soil conservation methods to evaluate the effect of precipitation and soil conservation measures on runoff and sediment load, but the measured data do not directly reflect the natural conditions of runoff derived from returning irrigation water in the ZRB, which hindered further studies of soil conservation planning and future water management policies. 

A catchment generally has a defined amount of runoff and sediment load under unchanged underlying surface conditions. Runoff and sediment load have a functional relationship and they are both influenced by precipitation and the underlying surface. A wide range of methods have been developed to quantitatively study the contribution of climatic and human activities in river hydrological processes (i.e., water) [30]. Water balance equations, hydrological models and soil conservation methods are all common methods to calculate the runoff and sediment discharge reduction effects. Of these methods, the hydrological models (such as correlation formulas and multiple regression methods) can effectively quantify precipitation-runoff-sediment relationships through the establishment of multiple regression equations [31] and construct intuitive and simple models with long-term series measure data in the large area. However, the soil conservation method requires detailed data such as the number and area of soil and water conservation practices, even small engineering conservation constructions (ponds, dam, flood lands and so on) and their runoff and sediment reduction benefits. Unfortunately, it’s difficult to collect these data and even harder to investigate in most watersheds. In addition, water balance equations require extensive observational data and hydrological parameters (i.e., precipitation, temperature, geology, soil, vegetation, and digital elevation models (DEMs)) [32]. Therefore, in the situation of calculating runoff and sediment variation in large areas, the hydrologic model method can get more appropriate results. In terms of identification of abrupt points, double mass curve (DMC) analyses are widely use methods which have relative merits of small data requirements and high transferability [33,34] and detect the abrupt change point with an initiate and simple way [35,36,37,38]. However, DMC analyses produce change points that are more or less subjective [39]. As to the development of methods for detecting abrupt points in recent years, a variety of hydrological factor diagnosis methods were proposed, such as the Pettitt, Mann-Kendall, accumulated variance analysis method [40], sequential cluster method, Lee-Heghinian method [41], and moving *t*-test technique [42,43] that can used to verify the accuracy of DMC analyses. 

Thus, in this study we take into account both runoff and sediment load change under climate change and soil and water conservation practices (mainly referring to vegetation measures and engineering measures), in a typical watershed in the middle of Yellow River where lots of soil and water conservation practice were implemented since the 1980s. The objectives of this study are as follows: (1) Analyze the change trends of the precipitation, runoff, sediment discharge and soil conservation measures during 1956–2015 in the ZRB; (2) estimate abrupt change points using DMC analyses and diagnostic hydrological methods; (3) illustrate the runoff and sediment discharge reduction effects of the soil conservation measures using various methods, including correlation formulas and multiple regression methods; (4) estimate the contribution rates of precipitation and soil conservation measures to runoff and sediment discharge. (5) characterize the influence of climate change and human activities on runoff and sediment discharge during 1956–2015 in the ZRB.

## 2. Materials and Methods 

### 2.1. Study Area Description

**Figure 1:**
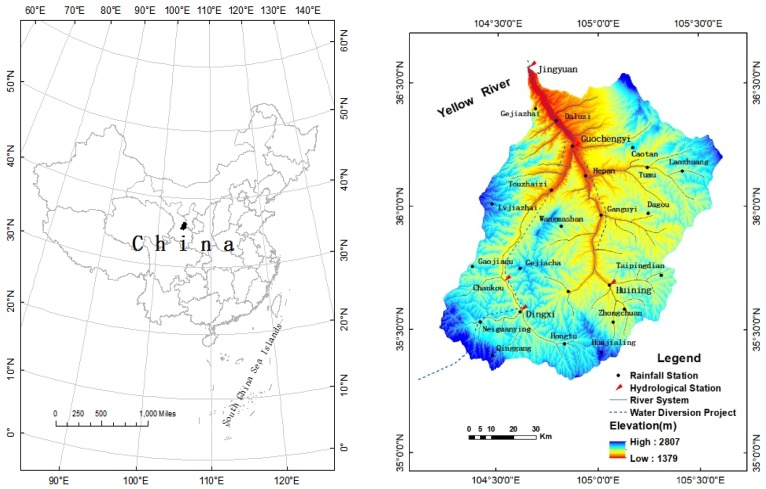
Location of the study area and distribution of stations in the Zuli River Basin.

The Zuli River, a tributary of the Yellow River, is located in the northwestern part of the Loess Plateau (104°13′~105°35′ E, 35°16′~36°34′ N), China (Figure 1). It flows through the six counties (Tongwei, Longxi, Huining, Anding, Yuzhong and Jingyuan) of Gansu Province and a portion of the Ningxia Hui Autonomous Region and finally flows into the Yellow River at Hongjuzi in Jingyuan County. The Zuli River Basin (ZRB) covers an area of 10,647 km^2^. The climate is arid and semi-arid, with warm summers and cold and dry winters [23]. The vegetation coverage is low and mainly consists of natural grass and irrigated vegetation, with few naturally distributed forest resources. The soil types are mainly black mound soil, calcareous soil and loess soil, and salinization is high in the basin. 

The intensive rainfall and the hilly terrain cause high soil erosion during flooding seasons on the Loess Plateau [44]. The average annual precipitation in the ZRB is 370.0 mm and is mostly concentrated from June to September. The average annual runoff and runoff depth are 1.129 × 10^8^ m^3^ and 10.6 mm, respectively, and the runoff coefficient is 0.029. The ZRB once had the highest annual erosion yield among the adjacent rivers in the upper reaches of the Yellow River [45]. The annual sediment yield in the basin is up to 5.0 × 10^6^ t, the annual erosion modulus is 4710 t·km^−2^ and the maximum sediment content can reach 1120 kg m^−3^.

### 2.2. Data Sources

This study mainly used the long-term hydro-meteorological and statistical data for soil conservation (Table 1). 

The precipitation, runoff, and sediment discharge data are controlled by national standards. The runoff and sediment data in 1956–2015 and rainfall data in 1971–2015 in this study are complete, and the missing data for a few rainfall stations were data of several rainfall stations in 1956–1970 and interpolated using neighboring stations’ data using a correlation method. To be specific, we built correlations between the missing station data and its adjacent hydrological stations’ data in 1971–2015, and then substituted adjacent hydrological stations data during 1956–1970 into the correlation to calculate the missing station data in 1956–1970. Thus the rainfall data of Hepan, Caotan, Ganguyi stations was interpolated by the long series of rainfall data of Guochengyi station. Dagou, Taipingdian stations interpolated by Huining station and Daluzi station interpolated by Jingyuan. These stations’ locations were then updated (Figure 1). After 1973, the returning water from irrigation led to an increase in runoff because the inter-basin water transfer project diverted water from the Yellow River to the ZRB. Therefore, this study used the natural runoff data calculated by an empirical coefficient of irrigation return water and the farmland water consumption coefficient of irrigation reported by the Gansu Water Resources Bulletin [46]. 

### 2.3. Abrupt Change Point Analysis

Double mass curve (DMC) analyses have been used to test the consistency of the relationships between two variables, analyze the trend of change for variables and determine abrupt change points in the same period [4]. In the study of runoff and sediment discharge in the Yellow River, abrupt change points are often detected by the turning points of curves or comparisons to watershed management activities [47]. Moreover, the hydrological variation diagnosis system used to test the abrupt change point based on the basic principles of methods (i.e., accumulated variance analysis, order cluster analysis, sliding *t*-test and Lee-Heghinian method) supported by the SharpDevelop platform developed by VB.Net. 

### 2.4. Correlation Formula

The correlation formula method is a useful statistical analysis method that can be used to construct comprehensive relationships between runoff (sediment discharge) and precipitation in the baseline period. What’s more it is a useful method to study the reduction effect of soil conservation measures on runoff and sediment discharge. Annual precipitation, flood season precipitation and maximum precipitation in 24 h are all factors to consider when choosing precipitation as an independent variable. The comparative analysis shows that the correlation coefficient of precipitation and runoff (sediment discharge) in flood season is the highest among the other factors. Therefore, this study chooses the flood season precipitation as the independent variable to construct the relationship during the baseline period as follows:

(1) $$W=\alpha_{1}\times P_{f}{}^{\beta_{1}}$$

(2) $$W_{s}=\alpha_{2}\times P_{f}{}^{\beta_{2}}$$

where *W* is the annual runoff; *Ws* is the annual sediment discharge; *P_f_* is the flood season precipitation. The components *α*_1_, *β*_1_, *α*_2_ and *β*_2_ are undetermined coefficients. 

### 2.5. Multiple Regression

This study divided the soil conservation measures into two categories: engineering measures and vegetation measures. Among them, the engineering measures mainly include terraces and sediment trapping dams, and the vegetation measures include tree plantations, grassland and closing management (referring to afforestation under the close hillsides). Regarding annual runoff (*W*) and annual sediment discharge (*Ws*) as dependent variables, the average annual precipitation (*P*), the area of engineering measures (*A_pro_*) and the area of vegetation measures (*A_veg_*) are used as independent variables in constructing the nonlinear multiple regression model as follows:

(3) $$W=K_{1}\times P^{m_{1}}\times A_{pro}{}^{n_{1}}\times A_{veg}{}^{l_{1}}$$

(4) $$W_{S}=K_{2}\times P^{m_{2}}\times A_{pro}{}^{n_{2}}\times A_{veg}{}^{l_{2}}$$

where *k*_1_, *m*_1_, *n*_1_, *l*_1_, *k*_2_, *m*_2_, *n*_2_ and *l*_2_ are undetermined coefficients.

The mean values of *P*, *A_pro,_* and *A_veg_* in the baseline period are then respectively substituted into the established multiple regression equations to obtain the runoff (*W_n_*) and sediment discharge (*Ws_n_*) under the impact of the average annual precipitation (*P*) and the soil conservation measures for the baseline period. The mean value of *P* in the measured period and the mean values of *A_pro_* and *A_veg_* in the baseline period are substituted into the equation to calculate the runoff (*W_p_*) and sediment load (*Ws_p_*) generated after the precipitation change. The decreased amounts of runoff and sediment discharge due to the change of precipitation are:

(5) $$\Delta W_{p}=W_{p}-W_{n}$$

(6) $$\Delta W_{S_{p}}=W_{S_{p}}-W_{S_{n}}$$

Similarly, the change of runoff and sediment discharge caused by the change of engineering measures and vegetation measures can be calculated separately, and the results are respectively indicated as Δ*W_pro_*, Δ*Ws_pro_*, Δ*W_veg_* and Δ*Ws_veg_*.

The relative contribution rates of precipitation for the changes in runoff and sediment discharge are then calculated using the following formulas:

(7) $$\eta_{P}=\Delta W_{P}/(\Delta W_{P}+\Delta W_{\mathrm{pro}}+\Delta W_{\mathrm{veg}})\times 100\%$$

(8) $$\eta_{S_{p}}=\Delta W_{S_{p}}/(\Delta W_{S_{p}}+\Delta W_{S_{\mathrm{pro}}}+\Delta W_{S_{\mathrm{veg}}})\times 100\%$$

Using the same pattern, the relative contribution rates of engineering measures and vegetation measures for changes in runoff and sediment discharge can be divided. The results are respectively designated as *η_pr_*, *ηs_pro_*, *η_veg_* and *ηs_veg_*.

## 3. Results

### 3.1. Variations of Hydrological Features

**Figure 2:**
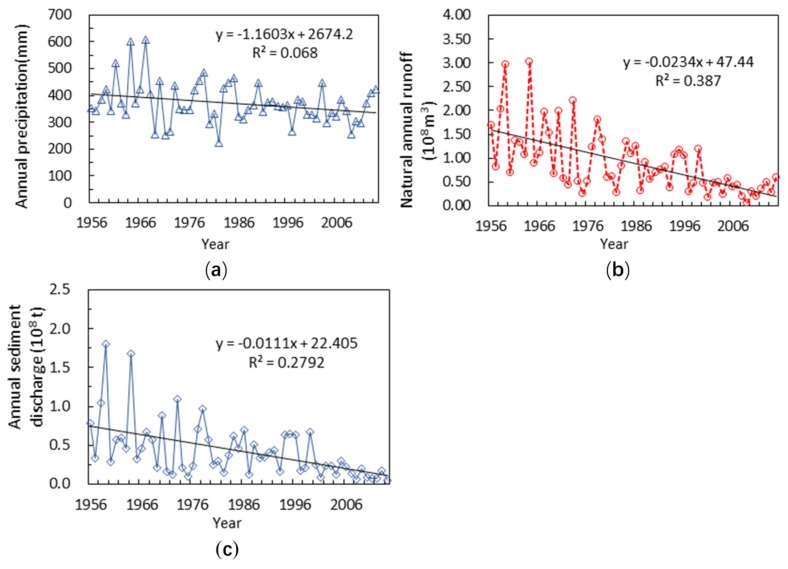
Evolution of precipitation (**a**), runoff (**b**) and sediment discharge (**c**) in the Zuli River Basin over the years 1956 to 2015.

Table 2 illustrates the characteristic of precipitation, runoff and sediment discharge during 1956–2015 in the ZRB. The annual average precipitation was 370.0 mm, and the annual runoff and annual sediment discharge were 0.8909 × 10^8^ m^3^ and 0.4232 × 10^8^ t, respectively. The precipitation show the same change trend as runoff and sediment discharge which all decreased from 1956–1979 to 1980–1989 and then increase and decrease in alternating changes after the 1980s. As shown in Figure 2, the annual average surface precipitation showed an increasing trend during 1956–1960. After this period, it showed a decreasing trend. The maximum annual runoff and annual sediment discharge occurred in the 1956–1979.

### 3.2. Changes in Soil Conservation Measures

**Figure 3:**
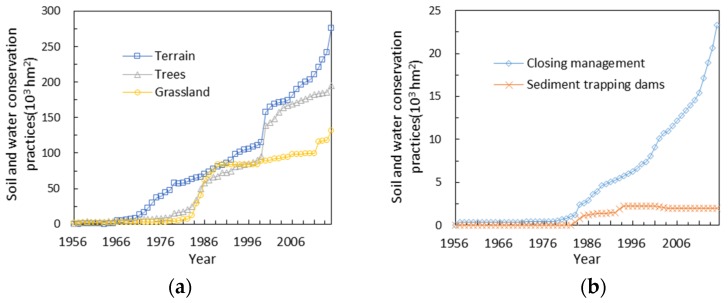
Temporal variations in the areas of soil and water conservation practices in the Zuli River basin over the years 1956 to 2015. (**a**) Description of Terrain, Trees, Grassland; (**b**) Description of Closing management and Sediment trapping dams.

The change in the area occupied by soil conservation measures is shown in Figure 3. The area in which soil conservation measures were implemented in the 1950s and 1960s was very small. After the 1970s, basic farmlands were vigorously constructed and the terrain increased substantially from 153 hm^2^ to 276.56 × 10^3^ hm^2^ from 1956 to 2015. The area occupied by sediment trapping dams increased after the 1980s, followed by a slow decreasing trend in 1990s. Vegetation measures, including tree plantations, grassland, and closing management, increased continuously, beginning in the 1980s. Closing management indicated a notable increasing trend from 160 hm^2^ to 23.31 × 10^3^ hm^2^ during 1978–2015. The areas occupied by tree plantations and grassland increased from 8.743 × 10^3^ hm^2^ and 3.426 × 10^3^ hm^2^ to 195.31 × 10^3^ hm^2^ and 131.46 × 10^3^ hm^2^, respectively, during 1978–2015.

### 3.3. Abrupt Change Point Analysis

**Figure 4:**
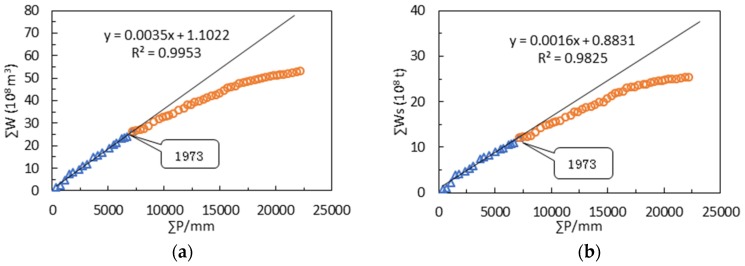
Double mass curves for annual runoff-precipitation (**a**) and annual sediment discharge-precipitation (**b**).

The double mass curves for annual runoff-precipitation and annual sediment discharge- precipitation during 1956–2015 are shown in Figure 4. The results demonstrate how the soil conservation measures impact runoff and sediment discharge. The abrupt change point appeared in 1973. 

**Figure 5:**
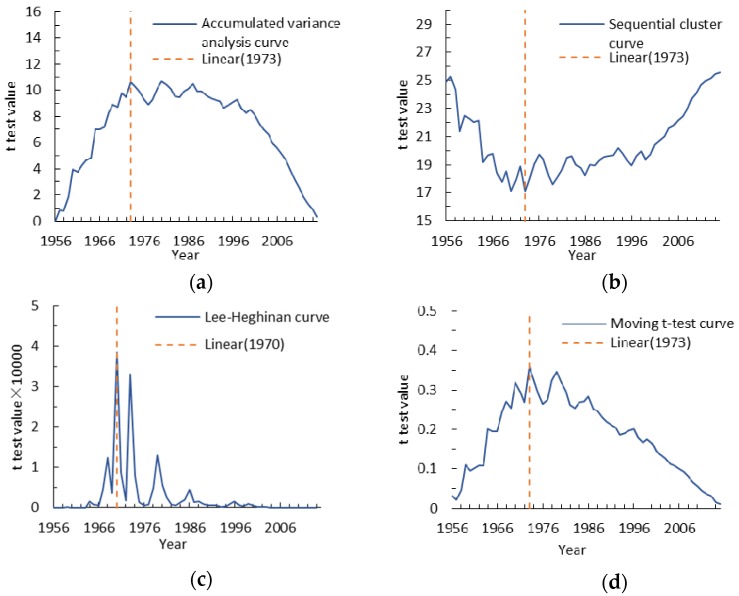
Analysis curves for the change point tested by hydrological variation diagnosis methods for annual runoff in the Zuli River Basin. (**a**) Description of the accumulated variance analysis curve for annual runoff in the first panel; (**b**) Description of sequential cluster curve for annual runoff in the second panel; (**c**) Description of Lee-Heghinan curve for annual runoff in the third panel; (**d**) Description of Moving *t*-test curve for annual runoff in the fourth panel.

**Figure 6:**
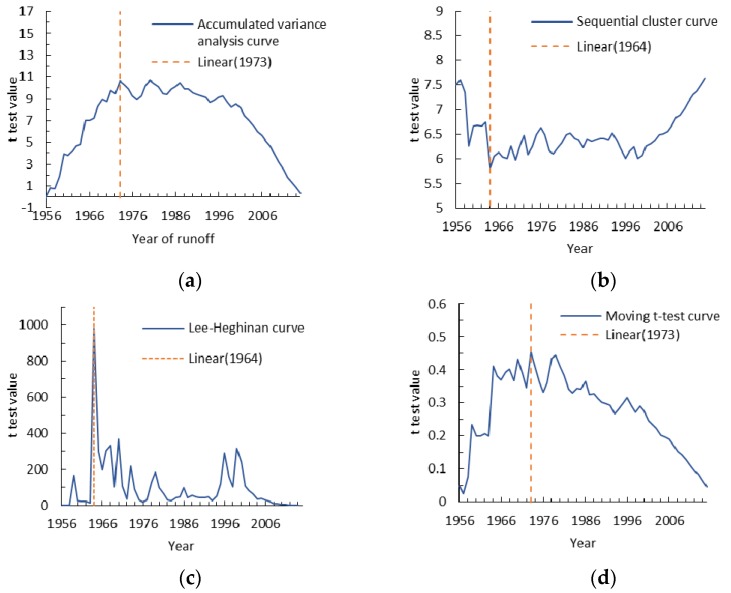
Analysis curves for the change point tested by hydrological variation diagnosis methods for annual sediment discharge in the Zuli River Basin. (**a**) Description of the accumulated variance analysis curve for annual sediment discharge in the first panel; (**b**) Description of sequential cluster curve for annual sediment discharge in the second panel; (**c**) Description of Lee-Heghinan curve for annual sediment discharge in the third panel; (**d**) Description of Moving *t*-test curve for annual sediment discharge in the fourth panel.

We chose four different hydrological variation diagnosis methods like accumulated variance analysis, sequential cluster, Lee-Heghinian and moving *t*-test method to further confirm the abrupt change point for annual runoff and sediment discharge identified by double mass curves (Figure 5 and Figure 6). The abrupt change points determined for annual runoff using above four different hydrological variation diagnosis methods are the years 1973, 1973, 1970 and 1973 (Figure 5).

The accumulated variance analysis method is determined by the long-term evolution and change trend based on the fluctuation of the accumulative variance value curve. The change point for runoff obtained by this method in this paper was 1973 because of the value of accumulative variance in this years was the highest (Figure 5a). Figure 5b shows that the minimum sum of deviation squares values calculated by sequential cluster method for runoff in the lowest position of curves is 1973. The abrupt point descripted by the Lee-Heghinan method was 1970 with a higher value of posteriori conditional probability density function f (Figure 5c). 

The moving *t*-test method separated a continuous sequence of hydrological meteorology into two different sub-sequences to determine whether the mean difference exceeds a certain level of significance. In this paper, we set the α equal 0.05, and found that the abrupt point show a distinct drift with the time window sliding in 1973. Similarly, the change points for annual sediment discharge are the years 1973, 1964, 1964 and 1973 (Figure 6). In 1964, the ZRB catchment was mainly affected by heavy rainfall, and one heavy rainstorm occurred in the middle and lower reaches in June. The maximum precipitation in 24 hours reached 131 mm, and annual precipitation for 1964 was 62.7% more than the annual average. The annual runoff and sediment discharge increased 189% and 296%, respectively, compared with their annual average values. Consequently, we can preclude the year of 1964 and choose the year 1973 as the abrupt change point and divide the entire period into the baseline period (1956–1973) and the measured period (1974–2015).

### 3.4. The Effect of Runoff and Sediment Reduction Caused by Soil Conservation Measures 

**Figure 7:**
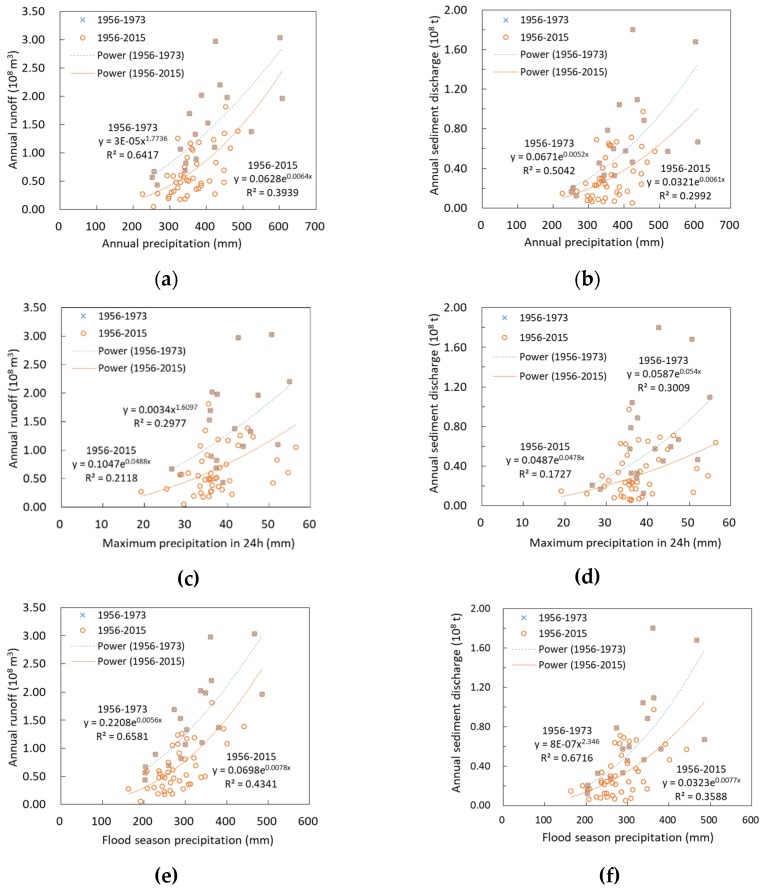
Related graphs of annual runoff (sediment discharge) -annual precipitation (**a**,**d**) and annual runoff (sediment discharge) -maximum precipitation in 24h (**b**,**e**) and annual runoff (sediment discharge) -flood season precipitation (**c**,**f**) in 1956–1973 and 1956–2015 in the Zuli River Basin.

The relationships between annual runoff (sediment discharge) and precipitation in the ZRB from 1956–1973 and 1956–2015 are shown in Figure 7. Annual precipitation, flood season precipitation and maximum precipitation in 24 h are all factors to consider when choosing precipitation as an independent variable. In the whole period, the correlation coefficient between three precipitation factors and runoff (sediment discharge) were all smaller than 0.5. However, the coefficient of three precipitation factors (order as annual precipitation, and maximum precipitation in 24 h and flood season precipitation) and annual runoff in the baseline period (1956–1973) were 0.642, 0.298 and 0.658, respectively, and between sediment discharge were 0.504, 0.301 and 0.672 separately. The comparative analysis shows that the correlation coefficient between three precipitation and annual runoff (sediment discharge) in the baseline period were higher than those in the whole research period. Besides, the correlation coefficient of precipitation and annual runoff in flood season is the highest among the other precipitation factors. The correlation coefficient between annual precipitation and annual runoff is slightly less than that in flood season and the correlation coefficient of the maximum 24h precipitation is lowest. The results are shown in Figure 7. Therefore, this study chooses the flood season precipitation as the independent variable to construct the relationship during the baseline period. 

The runoff and sediment discharge reduction effects from soil conservation measures are presented in Table 3. From 1974 to 2015, the reduction effects for runoff and sediment discharge were 45.5% and 32.5%, respectively, and the runoff reduction effect was greater than that of sediment discharge. As shown in Table 3, the changing trends of runoff reduction are in good agreement with those of sediment discharge, with an initial decreasing trend that changed to an increasing trend. The minimum values for the reduction of runoff and sediment discharge were 34.2% and 8.0%, respectively, in the 1990s, which were associated with the high amounts of precipitation and the apparent decrease in soil conservation measures in the 1990s.

### 3.5. Contribution Rate 

The multiple regression methods are used to evaluate the relationships between natural runoff, sediment discharge, average annual precipitation and the areas of engineering measures and vegetation measures during 1956—2015 in the ZRB as follows:

(9) $$W=0.000010161\times P^{2.0165}\times A_{pro}{}^{-0.0573}\times A_{veg}{}^{-0.1492}$$

(10) $$W_{S}=0.0000090667\times P^{1.8879}\times A_{pro}{}^{-0.0823}\times A_{veg}{}^{-0.1106}$$

It was found that the variation of runoff and sediment discharge was caused by precipitation changes, engineering measures and vegetation measures with large variabilities (Table 4 and Table 5). During 1974–1979, the volume of runoff and sediment discharge caused by precipitation change was increased because the annual average precipitation was 400.5 mm, which was higher than that in other periods. However, the precipitation in other periods was less than that in 1974–1979, which results in a decrease of runoff and sediment discharge. The runoff reduction for the entire period caused by the decrease in precipitation and the implementation of soil conservation measures was 0.232 × 10^8^ m^3^ and 0.207 × 10^8^ m^3^, respectively. The vegetation measures had a considerable impact, resulting in a decrease of 0.470 × 10^8^ m^3^ in runoff. The engineering measures reduced the runoff by 0.207 × 10^8^ m^3^. The sediment discharge decreased by 0.094 × 10^8^ t, which was caused by changes in precipitation. Soil conservation measures reduced the sediment discharge by 0.281 × 10^8^ t, of which 0.158 × 10^8^ t was caused by vegetation measures and 0.123 × 10^8^ t was caused by engineering measures. 

Table 5 shows the relative contribution rates of factors influencing runoff and sediment discharge reduction. The contribution rates of precipitation in the reduction of runoff and sediment discharge are approximately 25.5% and 25.0%, respectively. The contribution rates of vegetation measures are 51.6% and 42.2%, respectively, and are 22.8% and 32.8%, respectively, for engineering measures. As shown in Table 4, the precipitation in 1974–1979 increased compared with the baseline period, and the contribution rate of precipitation increased by 11.5% and 9.4%, as shown in Table 5. The primary soil conservation measures in the 1970s were the construction of terraces, with less development of areas of trees and grassland. Therefore, the contribution rate for engineering measures in the reduction of runoff and sediment discharge was greater than that for vegetation measures. Vegetation measures since the 1980s have included the rapid establishment of tree plantations and grassland; therefore, the contribution rate of vegetation measures since the 1980s has been greater than that of engineering measures. 

## 4. Discussion

**Figure 8:**
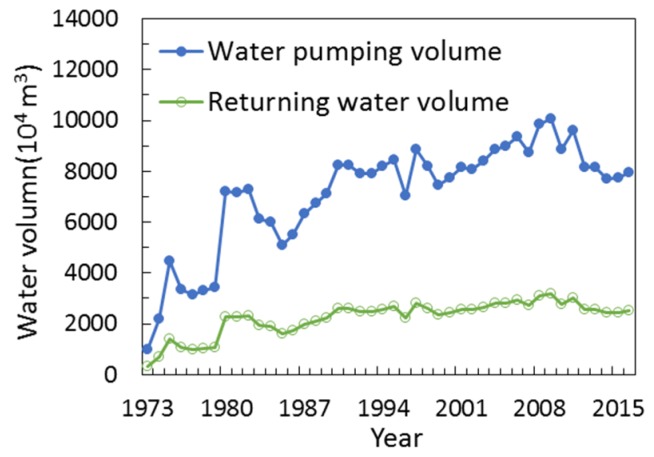
Water volume of the Yellow River irrigation project in the Zuli River Basin.

**Figure 9:**
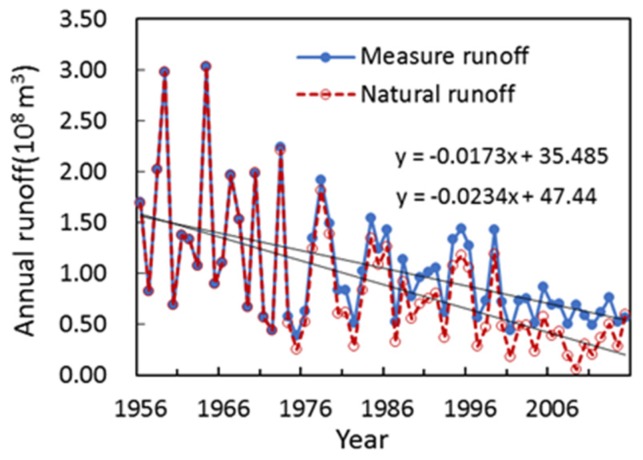
Graph comparing measures runoff and natural runoff.

The Zuli River Basin is a typical watershed in the middle of the Yellow River and its hydrological process and vegetation cover have changed greatly due to the large extent of soil and water conservation practices implemented by the Chinese government since 1980s. There is a direct influence of global climate change on water resource management and ecological environments [48]. The ZRB lies in the transitional zone between the semi-arid, sub-humid and arid climate zones and is one of the most sensitive, intense and complex areas of climate change in the complicated region of China. The precipitation in the ZRB is currently still in a low period and it has been reduced substantially in the past 50 years. The regional average precipitation has significantly impact on runoff and sediment discharge in the watershed. In 1964, annual precipitation reach 600.8 mm and much more larger than the annual average precipitation (just 370 mm) during 1956–2015 in ZRB which mainly affected by the extreme rainfall in the middle and lower reaches in June, 1964. Under these situation, the annual runoff and sediment discharge increased 189% and 296%, respectively. Besides, the annual precipitation and annual runoff (sediment discharge) showed a similar change trend in the whole research period (Table 2). That is to say, the volume of runoff and sediment discharge were closely related to precipitation changes in the ZRB. For example, the runoff and sediment discharge was increased 0.002 × 10^8^ m^3^ and 0.009 × 10^8^ t, respectively, during 1974–1979 due to the annual average precipitation higher than that in other period in measured period. Meanwhile, Zuli River Basin also called “the bitter river” because of its mineralization degree in midstream and downstream greater than 3 g/L and 10 g/L separately that mean humans and livestock all can’t use the water so the government started to launch a water transfer project outside the basin to resolve this problem. This project was built in 1971 and began operation in 1973 between Jingyuan County and Huining County and has a considerable effect on the surface water other than natural precipitation in the receiving area. The pumping capacity increased from 9.70 × 10^6^ m^3^ in 1973 to 0.78 × 10^8^ m^3^ in 2015 and there has been a cumulative pumping water volume of up to 0.30 × 10^10^ m^3^ since the project began operation. The irrigation area is mainly distributed in the middle and lower reaches of the ZRB. The water pumping and returning water volumes of the Yellow River irrigation project are shown in Figure 8 and measured runoff and natural runoff is presented in Figure 9. It is clear that the annual runoff data directly from the hydrologic gauges is not representative of natural runoff conditions after the construction of the irrigation projection in ZRB. The natural runoff data calculated with an empirical coefficient for irrigation return water was used herein to calculate the runoff reduction effect caused by soil conservation, and the results indicate that the runoff reduction effect is 45.5%, which is greater than the sediment reduction effect. Zhao [29] calculated the runoff reduction effect by using statistical methods and without considering the impact of the irrigation project on runoff reduction. He found that the runoff was reduced by 7.89 × 10^8^ m^3^, which accounted for 26.4% of the total runoff reduction. In our study, we used natural runoff which removed the impact of irrigation project on runoff to evaluate the runoff reduction effect. The result show that the reduction effect of runoff was 45.5% which less than the Zhao’s result approximately 19.1%. This difference is approximately the same as the current calculated volume of 0.22 m^3^ for irrigation return water, which accounted for 25.0% of the natural runoff. It proved that our results are not only correct but also can reasonable and reliable reflect the real situation of runoff change better in the Zuli River Basin through use the natural runoff. Besides, the relative contribution rates of climate and human activities influencing sediment discharge reduction calculated in our study were consistent with Xin [49] who chose the typical watershed in the middle of Yellow River to assess the effect of precipitation and human activities on sediment discharge, his results showed that the contribution rates of precipitation and human activities in the reduction of sediment discharge are approximately 20.3% and 79.7% separately in Kuye River Basin, 19.7% and 80.3% in Beiluo River Basin, 27.5% and 72.5% in Wuding River Basin. 

What’s more, the human activities have retarded or attenuated the impact on runoff and sediment discharge, particularly in the measured period with restoration of vegetation and construction of check-dams in the watershed [50]. Our results demonstrate that the runoff reduction effect is greater than the sediment discharge reduction effect through analyzing the influence of soil conservation measures on runoff and sediment discharge and is primarily due to two reasons: (1) The different allocation proportion of the soil conservation measures system has an important impact on reducing erosion and sediment discharge in the basin. The main flow-producing region of the ZRB is located upstream. For example, the runoff depths in the area above the Huining and Chankou stations are 12.9 mm and 12.2 mm, respectively, and the runoff depth in the middle and lower reaches of the ZRB is only 7.0 mm. The sediment discharge in the watershed occurs mainly in the middle and lower reaches, where the erosion modulus approaches 4205 t·km^−2^. The erosion modulus in each of the areas above the Huining and Chankou stations is only 3862 t·km^−2^ and 2913 t·km^−2^, respectively. The effect of soil conservation measures on runoff and sediment discharge began in the 1970s because of the vigorous construction of terraces in the 1970s and the implementation of vegetation measures in the 1980s, but the soil conservation measures are mainly concentrated in the upper reaches of the ZRB and there are relatively few measures that have been implemented in the middle and lower reaches of the river. Therefore, the runoff reduction effect of soil conservation measures analyzed in this study is greater than the sediment discharge reduction effect of soil conservation measures. (2) The conservation measures included vegetation measures (e.g., trees and grassland) and engineering measures (e.g., terraces and dams). The vegetation measures reduced the volume of precipitation during the flood period and can change streamflow through retention, penetration, absorption and transpiration of precipitation (Liu et al., 2014) and have a continuous soil conservation function. The engineering measures can capture and control surface runoff. However, the engineering measures are usually influenced by the quality of the terraces quality and warping of dams during their service lives. In this paper, the effects of vegetation measures on runoff and sediment discharge reduction are more notable than those of engineering measures. The use of vegetation measures resulted in a decrease of 0.470 × 10^8^ m^3^ for runoff and a decrease of 0.158 × 10^8^ t for sediment discharge from 1974 to 2015. However, the engineering measures reduced the runoff and sediment discharge by 0.207 × 10^8^ m^3^ and 0.123 × 10^8^ t, respectively, which are similar to the reduction of runoff and sediment discharge caused by precipitation. 

Therefore, from the standpoint of long-term management of soil conservation, vegetation measures are more effective than engineering measures and we should increase the construction and use of vegetation measures. Engineering measures are more effective if we want to control the sediment load of the Yellow River in a short period. 

## 5. Conclusions

The main objective of this study was to analyze the reduction effects of soil conservation measures on runoff and sediment discharge and to calculate the contribution rates of precipitation and soil conservation measures to runoff and sediment discharge during 1956–2015 in the Zuli River Basin. Flood season precipitation, natural runoff, and sediment discharge data from three main hydrologic stations were analyzed using the double mass curve and hydrological variation diagnosis methods. The results indicate that the abrupt change point for runoff and sediment discharge occurred in 1973. The runoff and sediment discharge reduction effect from soil conservation measures in the flood season was 45.5% and 32.5%, respectively, during the measuring period. In 1974–1979, the increase in precipitation led to contribution rates of 11.5% and 9.4% for runoff and sediment discharge, and the contribution rate of engineering measures to runoff and sediment discharge was greater than that of vegetation measures. After the 1980s, the contribution rate of vegetation measures was greater than the contribution rate of engineering measures. The contribution rate of vegetation measures to runoff and sediment discharge reached 50% and 40%, respectively. Therefore, this study recommends using the double mass curve and the hydrological variation diagnosis system to separate the entire study period into a baseline period and a measures period. Estimation of abrupt change point is a critical step for the analysis of the impact of precipitation and human activities on runoff and sediment discharge. More comprehensive studies are encouraged in the other watersheds of the Yellow River Basin. Meanwhile, this paper focuses on estimating the effect of climate and human activities on hydrological process through rough estimates on the large scale, but does not consider the process of transport because this involves lots of complex processes such as riverbank and gully erosion, interception by check-dams and vegetation and so on. In the future, we will pay more attention to this issue.

## Figures and Tables

**Table 1:** Description of data sources.

Sequence	Category	Period	Data Description
1	Precipitation	1956–2015	Monthly and yearly precipitation data from 22 rainfall stations provided by the Hydrology Department of Gansu Province
2	Runoff	1956–2015	Monthly and yearly runoff data from Jingyuan station provided by the Hydrology Department of Gansu Province
3	Sediment	1973–2015	Monthly and yearly sediment load data from Jingyuan station provided by the Hydrology Department of Gansu Province
4	Water conservancy project	1973–2015	Water pumping volume of Jinghui inter-basin water diversion project provided by Jinghui Electric Irrigation Engineering Authority of Baiyin, Gansu Province
5	Soil conservation measures	1973–2015	Area statistics for soil conservation measures
		2011	Soil conservation measures census results from Water Resources Department of Gansu Province

**Table 2:** Characteristic values of precipitation, runoff, and sediment discharge in different periods in the Zuli River Basin.

Series	Average Surface Precipitation		Runoff		Sediment Discharge	
	Annual (mm)	Coefficient of Variation (Cv)	Annual (10^8^ m^3^)	Coefficient of Variation (Cv)	Annual (10^8^ t)	Coefficient of Variation (Cv)
Average	370.0	0.20	0.8909	0.73	0.4232	0.84
1956–1979	409.5	0.24	1.4702	0.50	0.6932	0.64
1980–1989	353.2	0.20	0.7801	0.45	0.3823	0.47
1990–1999	364.8	0.12	0.7860	0.40	0.4313	0.45
2000–2009	332.1	0.15	0.3484	0.47	0.1863	0.40
2010–2015	350.6	0.15	0.3717	0.37	0.0870	0.44

**Table 3:** Runoff and sediment reduction effects caused by soil conservation measures.

Period	Runoff (10^8^ m^3^)			Sediment Discharge (10^8^ t)		
	Observed (10^8^ m^3^)	Simulated (10^8^ m^3^)	Reduction Effect (%)	Observed (10^8^ t)	Simulated (10^8^ t)	Reduction Effect (%)
1974–1979	0.9520	1.4783	35.6	0.4686	0.6292	25.5
1980–1989	0.7801	1.2266	36.4	0.3823	0.4977	23.2
1990–1999	0.7860	1.1947	34.2	0.4313	0.4688	8.0
2000–2009	0.3484	1.0079	65.4	0.1863	0.3778	50.7
2010–2015	0.3717	1.0943	66.0	0.0870	0.4242	79.5
1974–2015	0.6449	1.1840	45.5	0.3175	0.4705	32.5

**Table 4:** Reduction in runoff and sediment discharge due to variations in precipitation.

Period	Annual Precipitation (mm)	Annual Runoff (10^8^ m^3)^	Runoff Reduction (10^8^ m^3^)			Annual Sediment Discharge (10^8^ t)	Sediment Discharge Reduction (10^8^ t)		
			Precipitation	Engineering Measures	Vegetation Measures		Precipitation	Engineering Measures	Vegetation Measures
1956–1973	396.8	1.465				0.67			
1974–1979	400.5	0.952	0.1	0.126	0.002*	0.469	0.032	0.075	0.009*
1980–1989	353.2	0.78	0.384	0.173	0.255	0.382	0.127	0.103	0.103
1990–1999	364.8	0.786	0.47	0.197	0.19	0.431	0.158	0.117	0.076
2000–2009	332.1	0.348	0.517	0.229	0.368	0.186	0.175	0.135	0.149
2010–2015	350.6	0.372	0.535	0.244	0.269	0.087	0.182	0.143	0.109
1974–2015	357.3	0.645	0.47	0.207	0.232	0.317	0.158	0.123	0.094

* Represents an increasing amount; the other amounts are all decreasing amounts.

**Table 5:** The contribution rates for precipitation changes and soil conservation measures in the reduction of runoff and sediment discharge (%).

Year	Relative Contribution of Runoff Reduction			Relative Contribution of Sediment Reduction		
	Precipitation	Engineering Measures	Vegetation Measures	Precipitation	Engineering Measures	Vegetation Measures
1974–1979	11.5 *	62.1	49.4	9.5 *	76.9	32.6
1980–1989	31.5	21.3	47.2	30.9	30.8	38.3
1990–1999	22.2	23.0	54.8	21.8	33.2	45.0
2000–2009	33.0	20.6	46.4	32.4	29.4	38.2
2010–2015	25.7	23.3	51.0	25.1	33.0	41.9
1974–2015	25.5	22.8	51.7	25.0	32.8	42.2

* Represents an increasing amount; the other amounts are all decreasing amounts.
